# Cultivation of the causative agent of human neoehrlichiosis from clinical isolates identifies vascular endothelium as a target of infection

**DOI:** 10.1080/22221751.2019.1584017

**Published:** 2019-03-22

**Authors:** Linda Wass, Anna Grankvist, Lesley Bell-Sakyi, Malin Bergström, Erik Ulfhammer, Christine Lingblom, Christine Wennerås

**Affiliations:** a Department of Infectious Diseases, Institute of Biomedicine, Sahlgrenska Academy, University of Gothenburg, Göteborg, Sweden; b Department of Clinical Microbiology, Sahlgrenska University Hospital, Göteborg, Sweden; c Department of Infection Biology, Institute of Infection and Global Health, University of Liverpool, Liverpool, UK; d The Wallenberg Laboratory for Cardiovascular Research, Sahlgrenska Academy, University of Gothenburg, Göteborg, Sweden

**Keywords:** *Candidatus* Neoehrlichia mikurensis, endothelium, tick cell lines, circulating endothelial cells, neoehrlichiosis

## Abstract

*Candidatus* (*Ca*.) Neoehrlichia mikurensis is the cause of neoehrlichiosis, an emerging tick-borne infectious disease characterized by fever and vascular events. The bacterium belongs to the *Anaplasmataceae*, a family of obligate intracellular pathogens, but has not previously been cultivated, and it is uncertain which cell types it infects. The goals of this study were to cultivate *Ca.* N. mikurensis in cell lines and to identify possible target cells for human infection. Blood components derived from infected patients were inoculated into cell lines of both tick and human origin. Bacterial growth in the cell cultures was monitored by real-time PCR and imaging flow cytometry. *Ca.* N. mikurensis was successfully propagated from the blood of immunocompromised neoehrlichiosis patients in two *Ixodes spp*. tick cell lines following incubation periods of 7–20 weeks. Human primary endothelial cells derived from skin microvasculature as well as pulmonary artery were also susceptible to infection with tick cell-derived bacteria. Finally, *Ca.* N. mikurensis was visualized within circulating endothelial cells of two neoehrlichiosis patients. To conclude, we report the first successful isolation and propagation of *Ca.* N. mikurensis from clinical isolates and identify human vascular endothelial cells as a target of infection.

## Introduction

*Candidatus* (*Ca*.) Neoehrlichia mikurensis earned its name in 2004, when it was discovered in ticks and wild rodents collected and investigated on the Japanese island of Mikura between 1998 and 2003 [1]. This tick-borne agent had actually been described previously under other names including *Ehrlichia* “Schotti-variant,” *Ehrlichia*-like agent and *Ehrlichia walkerii* [2]. In Europe, it is one of the commonest human-pathogenic microbes carried by *Ixodes ricinus* ticks, after *Borrelia burgdorferi* sensu lato and *Rickettsia spp*. [2]. The bacterium gained renewed attention in 2010 when several case reports revealed its capacity to cause human disease [3–5]. This new infectious disease was named neoehrlichiosis and severe cases typically featured high fever with thromboembolic or vascular complications such as deep vein thrombosis, pulmonary embolism, transitory ischaemic attacks and arterial aneurysms [2,4]. Initially, *Ca.* N. mikurensis was believed to be an opportunistic bacterium that exclusively afflicted immune-suppressed patients with particular haematologic or autoimmune diseases [6]. However, persons with normal immune defense can also become infected by this new pathogen; the clinical picture among immune-competent individuals encompasses asymptomatic infections, skin rashes resembling *erythema migrans*, systemic infection with fever, and even a possible fatality from vascular complications [5,7–10].

Many, if not most, cases of neoehrlichiosis go unrecognized because the bacterium cannot be detected by routine microbiological diagnostic methods such as blood culture. This member of the family *Anaplasmataceae* is thought to be an obligate intracellular bacterium and consequently does not grow on cell-free culture media. The infection is often designated as “fever of uncertain origin” among immune-suppressed patients and any ensuing thromboembolic or vascular complications are misinterpreted as being age-related or due to other associated medical conditions, since the majority of patients are middle-aged or older with underlying diseases [6,11]. Currently, panbacterial or specific PCR of blood samples is the only means of diagnosis. There are no serological methods available since there are no cultured bacterial extracts for use in the development of ELISA or cell-based indirect fluorescence antibody assays. Lack of an *in vitro* culture system for *Ca.* N. mikurensis additionally hampers research on the pathogenic mechanisms of this new infectious agent, including the sequencing of its genome. An additional difficulty is that the natural target cells for infection by *Ca.* N. mikurensis are unknown. Structures resembling bacteria of the family *Anaplasmataceae* have been identified inside splenic sinusoidal endothelial cells of experimentally infected rats [1] and human neutrophilic granulocytes collected from an infected patient [12], but labelling these bacteria by antibodies or DNA probes was not attempted [1,12]. Furthermore, as both of these cell types belong to the reticulo-endothelial cell system and efficiently ingest noxious material, presence within them of bacteria could reflect efficient cellular immune defense rather than actual infection. Moreover, it should be borne in mind that since rodents infected by *Ca.* N. mikurensis do not appear to develop disease [2], and the splenic sinusoidal endothelium of rats differs markedly from that of humans [13], the cellular tropism of this microorganism may not be the same in rats and humans.

The objective of this study was the successful isolation and *in vitro* cultivation of *Ca.* N. mikurensis, and if possible, identification of the target cells for infection in humans. To this end, blood samples from neoehrlichiosis patients were inoculated into a variety of cell lines of tick and human origin.

## Results

### Successful propagation of infection from patient blood but not from ticks in tick cell lines

We first inoculated the tick cell lines IRE/CTVM20 and ISE6 with haemolymph or homogenates prepared from *Ca.* N. mikurensis-infected ticks that were collected by flagging. Tick cell lines derived from *I. ricinus* and *Ixodes scapularis* were selected because the former tick species is known to be a vector of *Ca.* N. mikurensis [2], and cells of the latter species support growth of the closely related *Neoehrlichia lotoris* [14,15]. However, despite 14 attempts and intermittent use of Amphotericin B, one-third of the cultures were lost to fungal contamination and infection was not transferred from any of the infected tick specimens to the tick cell lines (data not shown).

**Figure 1.**
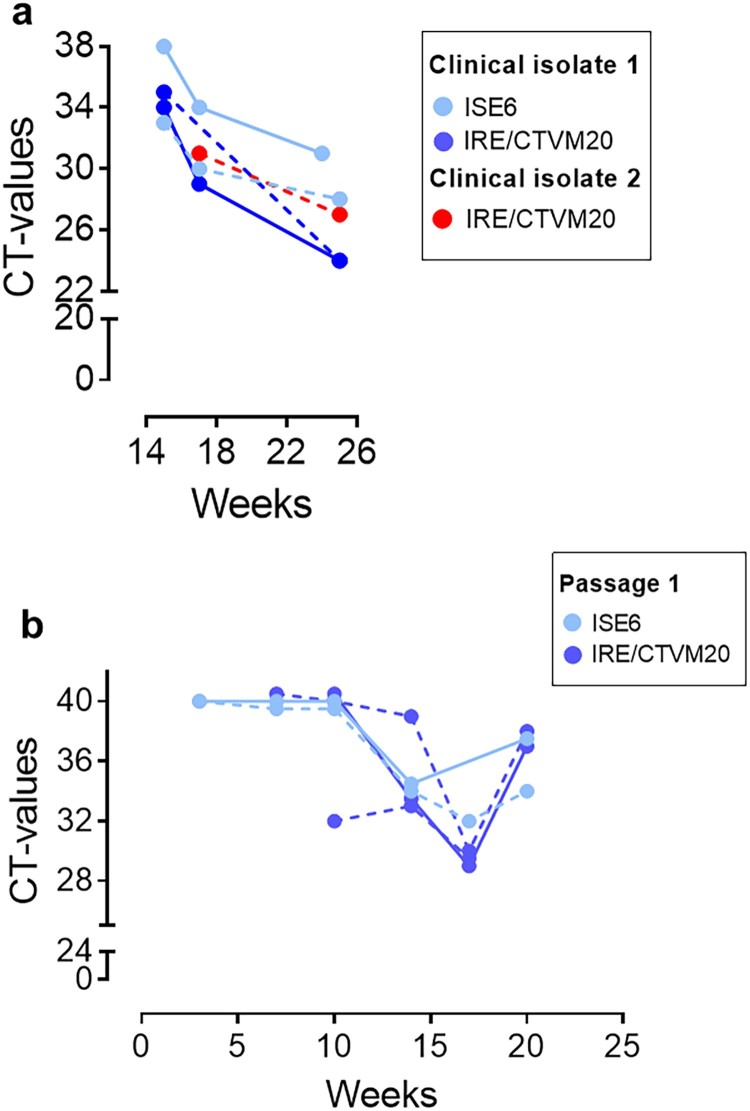
Isolation of *Ca.* N. mikurensis from patient blood into tick cell lines and passage of the infection. (a) Diminishing Cycle threshold (CT) values of *Ca*. N. mikurensis PCR amplicons in tick cell lines derived from *I. ricinus* (IRE/CTVM20) and *I. scapularis* (ISE6) inoculated with either whole blood (continuous lines) or plasma/buffy coat specimens (dashed lines) from two patients with neoehrlichiosis (SE15; blue symbols, and SE17; red symbols, Table 1). PCR results from undiluted tick cell extracts are shown. (b) CT values following passage of the infection (isolate SE15) from infected tick cell lines ISE6 and IRE/CTVM20 to uninfected homologous tick cell lines.

In contrast, we were able to transmit the infection from blood samples from six individual neoehrlichiosis patients (Table 1) to one or both tick cell lines. The kinetics of the infection were monitored by real-time PCR, and decreasing CT-values indicative of increasing amounts of bacterial DNA were apparent after 7–20 weeks of culture (Table 1); results from two representative patients (SE15 and SE17) are shown in Figure 1. The *I. ricinus* and *I. scapularis* cell lines seemed to be equally susceptible to infection, and unfractionated whole blood samples and buffy coat supplemented with plasma were equally good infectious material (Figure 1(a–b)). Importantly, passage of the infection to new uninfected tick cells was achieved for five of the clinical isolates, for example SE15, in which it may be seen that the CT-values began to decrease earlier already after 10 weeks following subculture (Figure 1(b)) compared with the initial culture (Figure 1(a)). Moreover, we succeeded in maintaining this first isolate in continuous culture through three passages over a period of 10 months.

**Table 1.** Clinical data of patients whose blood samples were included in the study.

Patient ID	Age	Sex	Disease	Immune suppression	Fever	Other symptoms	Vascular event	Tick bite	Infectious material	Cell line supporting growth of Neoehrlichia	First positive culture	Infected CEC detected	Ref.
SE15	57	F	Multiple sclerosis	Rituximab	1.5 months	Swollen foot, localized myalgia (trapezius muscle), bilateral pain lower legs upon exertion	Thrombo-phlebitis	Yes	Whole blood and plasma/buffy coat	IRE/CTVM20 ISE6	15 weeks	NA	[43]
SE17	58	M	Follicular lymphoma	Rituximab Chemotherapy	2 months	Myalgia upper and lower extremities, arthralgia both ankles	Repeated thrombo-phlebitis	Yes	Plasma/buffy coat	IRE/CTVM20	17 weeks	NA	[11]
SE21	63	F	Primary hypogamma-globulinemia (IgG1)	Splenectomy	1–2 weeks	Pain, sore palpation right flank and lower abdominal quadrant	No	Yes	Whole blood and plasma/buffy coat	ISE6	12 weeks	NA	[11]
SE23	48	F	Multiple sclerosis	Rituximab	4 months	Localized myalgia (left upper arm, left lower leg), arthralgia right wrist, swollen ankles, cough	Repeated muscle vein thromboses	No	Whole blood and plasma/buffy coat	IRE/CTVM20	20 weeks	Yes	This study
SE24	54	M	Granulomatosis with polyangiitis	Rituximab Chemotherapy IL-1R inhibitor Corticosteroids	1.5 years	Severe fatigue, skin rash, general myalgia, cough	DVT lower extremity	Yes	Whole blood and plasma/buffy coat	IRE/CTVM20 ATCC PCS-110-010 ATCC PCS-100-022	Tick cells: 9 weeks Endothelial cells: 15 days	NA	This study
SE25	62	M	Multiple sclerosis	Rituximab	2 months	Cough	No	Yes	Plasma/buffy coat	IRE/CTVM20 ATCC PCS-110-010 ATCC PCS-100-022	Tick cells: 7 weeks Endothelial cells: 6 days	NA	This study
SE26	65	M	B-Chronic lymphocytic leukemia	Rituximab Chemotherapy	6 days	Localized myalgia (neck), headache, cough	DVT upper extremity	No	NA	NA*	NA*	Yes	This study

Note: F = female, M = male, NA = not assessed, NA* = not assessed because of insufficient material, CEC = circulating endothelial cells.

The presence of penicillin and streptomycin in the tick cell culture medium did not appear to inhibit growth of *Ca.* N. mikurensis, as also seen with the closely related *Ehrlichia ruminantium*, the agent of the infectious disease heartwater of ruminants [16], and *Ehrlichia minasensis*, a tick-borne pathogen of cattle in Brazil [17]. Whether or not the antibiotics might have delayed initial establishment and spread of the bacteria in the tick cells is unclear, but long incubation periods on first infection of tick cells using mammalian stage *Anaplasmataceae* have been reported previously both with [16] and without [18] antibiotics, suggesting that low amounts of viable infective bacteria in the inoculum and/or a requirement for bacterial adaptation to the very different environment of a tick cell may be important contributory factors.

**Figure 2.**
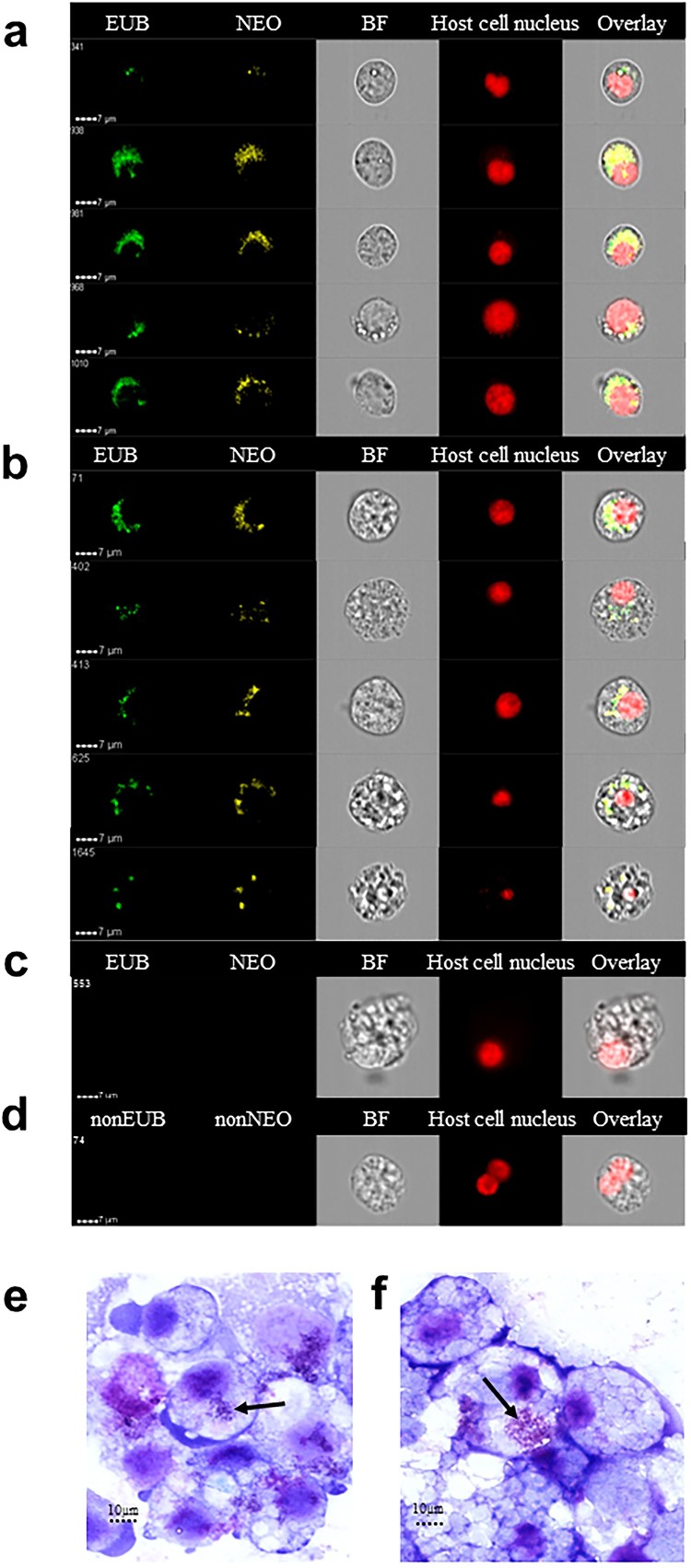
Visualization of *Ca.* N. mikurensis infection of tick cell lines. (a) Image flow cytometer depiction of *I. scapularis* ISE6 cells 9 weeks after the first passage of the infection, which originally had been maintained for 25 weeks of culture after inoculation with an infected blood sample (SE15). The cells were labelled using a panbacterial DNA probe (EUB) in green and a *Ca.* N. mikurensis-specific DNA probe (NEO) in yellow; bright field image (BF); red staining (DRAQ5) of the host cell nucleus (tick). Overlay image of all stains shows complete congruence of bacteria in the cytoplasm labelled using the panbacterial and *Ca.* N. mikurensis probes. (b) Panels as in (a) using *I. ricinus* IRE/CTVM20 cells inoculated with infected blood from patient SE15. (c) Mock-infected IRE/CTVM20 cells stained using the *Ca.* N. mikurensis-specific DNA probe (NEO), the panbacterial DNA probe (EUB) and host cell nucleus stain DRAQ5. (d) No hybridization signal was seen when infected IRE/CTVM20 cells were incubated with the control probes non-EUB338 or non-Neo. (e)–(f) Giemsa-stained cytocentrifuge smears of tick cells infected *in vitro* with *Ca.* N. mikurensis. (e) *I. scapularis* ISE6 cell line. (f) *I. ricinus* IRE/CTVM20 cell line. Arrows indicate bacterial inclusions.

To ensure the specificity of our findings, infected tick cells were labelled with fluorescent panbacterial and *Ca*. N. mikurensis-specific DNA probes, and analysed using image flow cytometry, a technique that combines flow cytometry and high resolution microscopy, enabling detailed visualization of individual cells. It was found that the tick cells inoculated with patient buffy coat admixed with plasma harboured cytoplasmic particles that were stained with the *Ca.* N. mikurensis probe. Varying patterns of staining were seen – ranging from a few individual rounded bacteria-like structures to densely packed groups of bacteria (Figure 2(a–b)). *Ca.* N. mikurensis tended to localize in what appeared to be cytoplasmic inclusions adjacent to the tick cell nucleus, which is exactly the same location that has been reported for its closest relative, *N. lotoris* of raccoons [14]. The bacteria that were stained by the *Ca*. N. mikurensis probe were also stained by the panbacterial probe as indicated by overlay images (Figure 2(a–b)). No bacteria were only stained by the panbacterial probe and not by the *Ca*. N. mikurensis-specific probe, indicating that there were no other bacterial contaminants present in the tick cells (Figure 2(a–b)). Importantly, no fluorescent staining was evident in mock-inoculated tick cells (Figure 2(c)), nor did complementary control probes give rise to non-specific hybridization (Figure 2(d)). The per cent of cells that were infected by *Ca.* N. mikurensis as estimated by using image analysis algorithms was 81% for the *I. ricinus* tick cell line versus 43% of the *I. scapularis* cell line after 8 weeks of culture. Giemsa-stained smears of the infected tick cell lines showed bacterial aggregates within the cell cytoplasm (Figure 2(e–f)), reminiscent of the *morulae* seen in cells infected by *Anaplasma phagocytophilum* or *Ehrlichia chaffeensis* [19,20].

### Successful transfer of infection from tick cell lines into human endothelial cells

**Figure 3.**
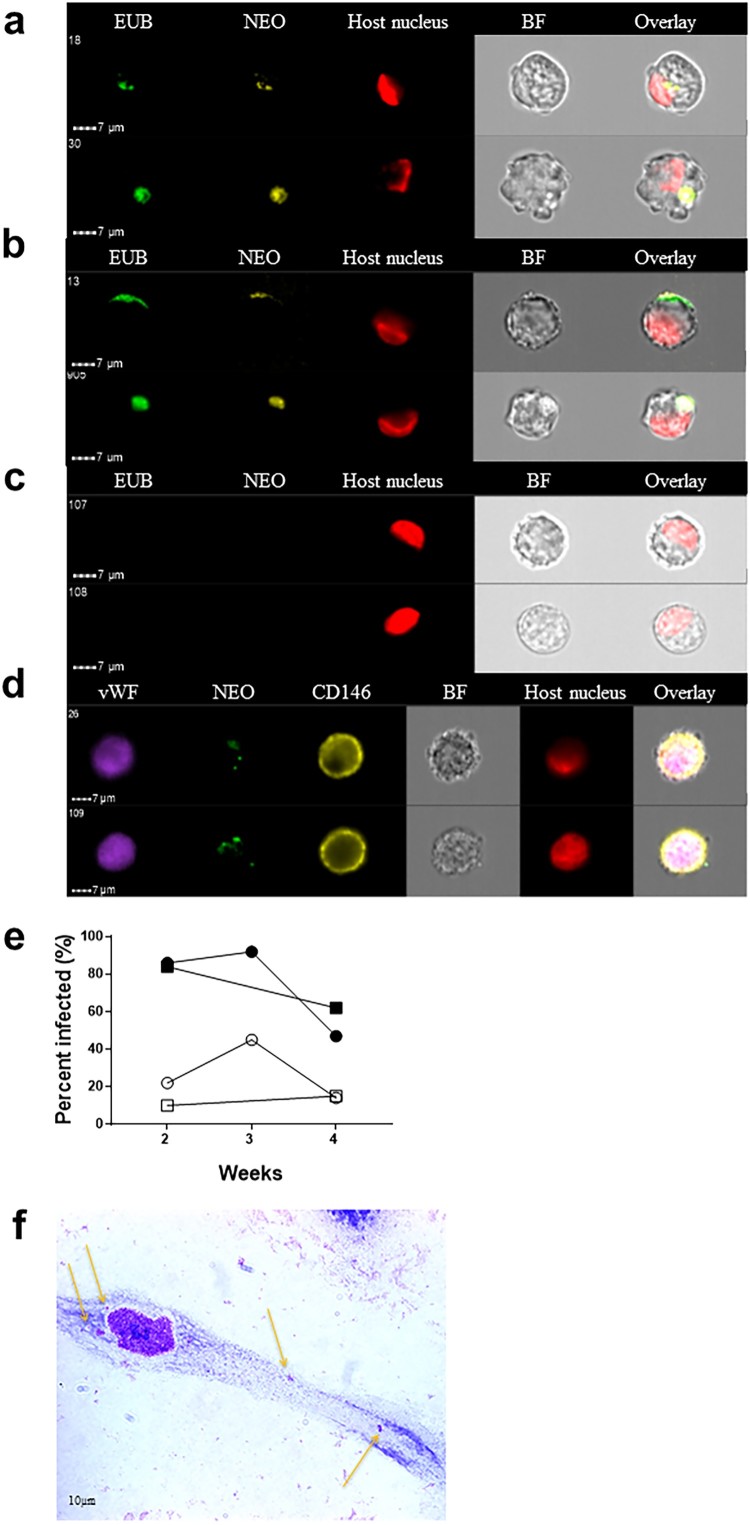
Visualization of *Ca.* N. mikurensis infection of primary endothelial cell lines. (a)–(b) Image flow cytometer depiction of endothelial cells from pulmonary artery 7–14 days after inoculation with homogenized tick cells that had been infected from clinical isolate SE25. The endothelial cells were labelled using a panbacterial DNA probe (EUB) in green and a *Ca.* N. mikurensis-specific DNA probe (NEO) in yellow; red staining (DRAQ5) of the host cell nucleus (endothelium), after (a) 1 week and (b) 2 weeks of culture. (c) Uninfected negative control endothelial cells did not stain with the EUB388 probe nor with the Neo probe. (d) Verification that the infected cells were endothelial by labelling them with a mAb against the von Willebrand factor (vWF, purple), the *Ca.* N. mikurensis-specific DNA probe (NEO, green), a mAb against CD146 (yellow), and staining of the endothelial cell nucleus (DRAQ5, red). Bright field images (BF) and overlay images are shown. (e) Graph illustrating the percentage of the two endothelial cell lines that were infected by *Ca.* N. mikurensis (⬤ = pulmonary artery endothelial cells, ▪ = skin microvasculature endothelial cells) and the fraction of the cytosol of these respective cell lines that were occupied by bacteria (○ = pulmonary artery endothelial cells, □ = skin microvasculature endothelial cells), after 2, 3 and 4 weeks of culture. (f) Giemsa-stained preparation of a cutaneous microvasculature endothelial cell infected *in vitro* by *Ca.* N. mikurensis. Arrows indicate bacterial inclusions.

After several unsuccessful attempts to directly infect endothelial cells with either tick extracts or infected patient blood, we attempted to transfer the infection from the infected tick cell lines to the endothelial cell lines. This strategy has previously proven to be successful for other members of the family *Anaplasmataceae*, including *Anaplasma marginale* and *A. phagocytophilum* [21]. Whereas it took 4–5 months for a *Ca.* N. mikurensis infection to establish in the tick cell lines, transfer of the infection from tick cells infected with bacteria originating from two different patients to the endothelial cells was evident already after one week (Figure 3(a)) and reached a peak after 2–3 weeks of infection (Figure 3(b)). No staining of uninfected control endothelial cells was seen using either of the two probes (Figure 3(c)). It was ascertained that these cell lines were indeed of endothelial origin and not fibroblasts or smooth muscle cells since they expressed the typical endothelial markers CD146 and von Willebrand factor (Figure 3(d)).

According to image analyses, more than 80% of the cutaneous microvasculature endothelial cells and the pulmonary artery endothelial cells contained bacteria after 2 weeks of culture (Figure 3(e)). The endothelial cell lines could only be maintained for a maximum of four weeks, by which time they were very damaged by the infection. The bacterial inclusions within cutaneous endothelial cells were also visualized using Giemsa stain: a single microvascular skin endothelial cell experimentally infected with tick cell-derived *Ca.* N. mikurensis showing low-level infection after 2 weeks of culture is depicted (Figure 3(f)).

**Figure 4.**
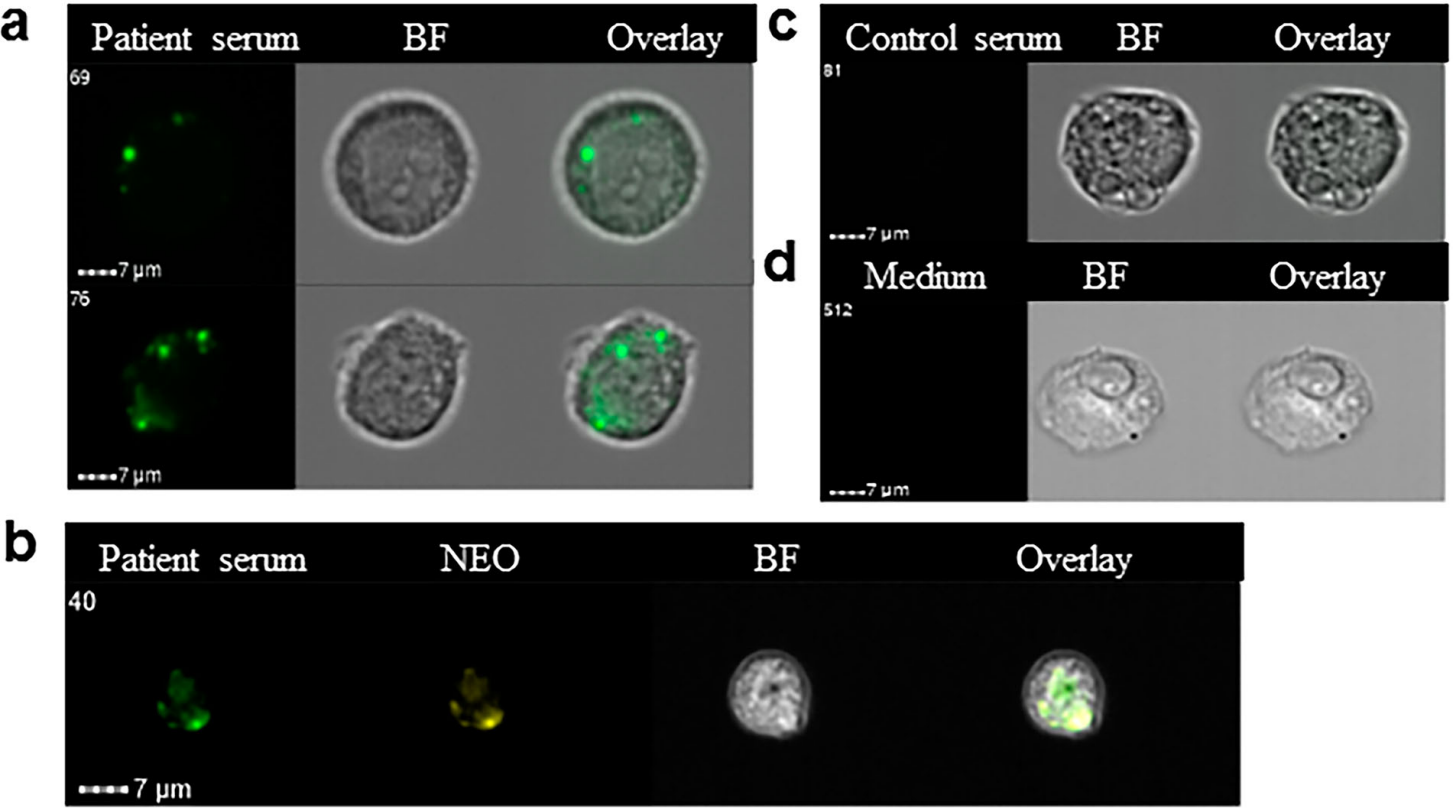
Immune serum stains bacterial inclusions in an endothelial cell line infected *in vitro* using a clinical isolate of *Ca.* N. mikurensis. (a) Image flow cytometer depiction of two infected endothelial cells incubated with serum from a patient with neoehrlichiosis (SE13), followed by a secondary FITC-labelled anti-human mAb. (b) The immune serum stains the same inclusions in endothelial cells as the *Ca.* N. mikurensis-specific probe. Bright field images (BF) and overlays are also shown. (c) Representative images show that the infected endothelial cells were not stained using control serum from a healthy individual or culture medium, followed by the secondary antibody.

As further corroboration of the specificity of the labelling of bacteria inside endothelial cells, immune labelling was done using immune serum from a neoehrlichiosis patient. It may be seen that bacterial inclusions were brightly stained using this serum (Figure 4(a)), and that the *Ca.* N. mikurensis-specific DNA probe and the immune serum bound to the same structures within the endothelial cell (Figure 4(b)). Neither a control serum from a healthy individual, nor culture medium alone followed by the secondary anti-IgG antibody (Figure 4(c)), stained the infected endothelial cells.

### Detection of *Ca.* N. mikurensis inside circulating endothelial cells of infected patients

**Figure 5.**
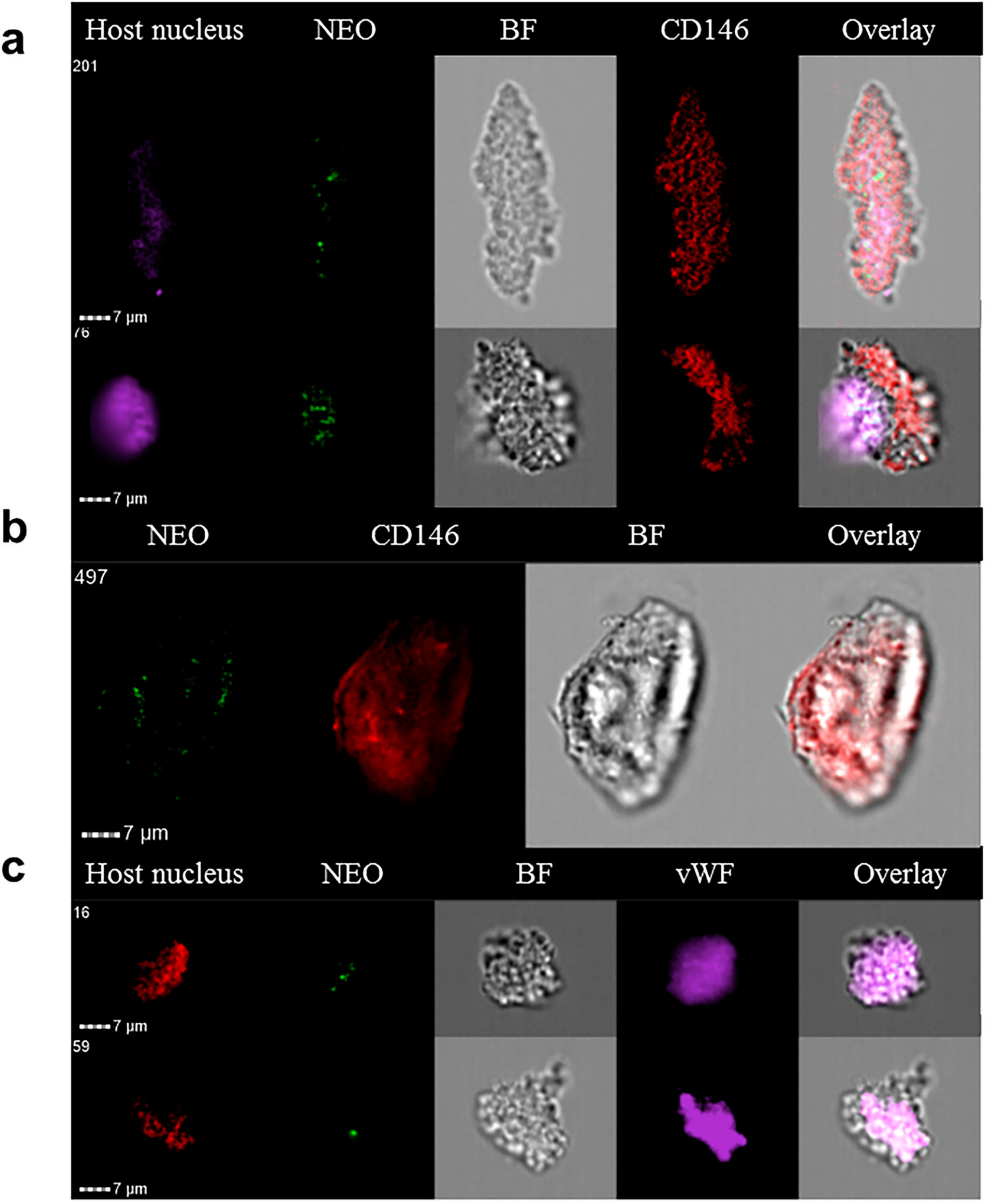
Circulating endothelial cells isolated from the blood of a neoehrlichiosis patient stain positive for *Ca.* N. mikurensis. Image flow cytometer depiction; the cells (derived from patient SE26) were stained using the *Ca.* N. mikurensis-specific DNA probe (NEO, green), DAPI or DRAQ5 (host cell nucleus, purple or red), and either the mAb anti-CD146 (red) or the mAb anti-von Willebrand factor (vWF, purple). Bright field image (BF) and overlays are shown for five different endothelial cells.

We hypothesized that it might be possible to detect circulating endothelial cells infected by *Ca.* N. mikurensis in the blood of neoehrlichiosis patients. Endothelial cells can be detected in extremely low numbers in the blood of healthy individuals [22]. Freshly isolated buffy coats from patients with newly diagnosed neoehrlichiosis were examined for the presence of large cells expressing CD146 and the von Willebrand factor, and containing *Ca.* N. mikurensis bacteria, by image flow cytometry. Indeed, such cells were identified in two patients (Table 1), establishing that *Ca*. N. mikurensis are found within endothelial cells in naturally infected humans (Figure 5). The infected cells that we identified were very large and elongated, measuring up to 50 µm in length, and appeared damaged, which is to be expected of dying cells infected by an intracellular pathogen (Figure 5). Similarly, detection of the agent of Mediterranean spotted fever, *Rickettsia conorii*, in circulating endothelial cells that were pulled out of the blood of an infected patient using magnetic beads coated with monoclonal antibodies to CD146, was reported to be difficult: the cells appeared as ghost cells with unclear cellular boundaries and pycnotic nuclei [23,24].

## Discussion

The severe form of neoehrlichiosis that mainly afflicts immune-compromised patients is associated with a very high incidence of vascular incidents [2], as reflected by the fact that two-thirds of the patients from whose blood samples we were able to cultivate *Ca*. N. mikurensis in this study suffered from venous complications. This raised the question as to whether these vascular events were the result of infectious vasculitis, which indeed seems to be possible since we were able to infect endothelial cell lines with clinical isolates of *Ca.* N. mikurensis. A number of arguments favour the endothelial cell as being a target cell of human infection by *Ca.* N. mikurensis: the suspected infection of splenic rat endothelial cells by the bacterium [1], the vascular events afflicting neoehrlichiosis patients that could indicate endothelial infection [6], and the close genetic relationship of *Ca.* N. mikurensis with *E. ruminantium* which infects the vascular endothelium [25,26].

Endothelial cells are heterogeneous and vary both functionally and phenotypically depending on their origin, e.g. arterial or venous, size of the blood vessel and in which organ or part of the body [27]. We chose human endothelial cells derived from the skin microvasculature to infect with *Ca.* N. mikurensis for obvious reasons – the first contact of the bacterium with the endothelium is likely to occur in the skin following its transmission via a tick bite. A primary pulmonary arterial endothelial cell line was also selected because there are two published case reports of neoehrlichiosis associated with the development of arterial aneurysms, one of which afflicted the pulmonary artery [5,6].

Final evidence for the vascular endothelial hypothesis was the demonstration of the bacteria within what are termed “circulating endothelial cells.” There are two main types of endothelial cells in the blood circulation, circulating endothelial cells and endothelial progenitor cells. Whereas the circulating endothelial cells are believed to be fully mature cells that have detached from the vessel wall, either as part of the normal turn-over of blood vessels or due to damage to the vessel wall, endothelial progenitor cells are thought to be endothelial precursor-like cells released from the bone marrow whose function is to repair injured vessels [22]. Although there is no clear consensus on the exact molecular marker profiles that define these two types of endothelial cells, the circulating endothelial cells are generally defined as large cells (10–50 µm in diameter) that express CD146 on the surface and the von Willebrand factor intracellularly [22,28]. In contrast, endothelial progenitor cells are smaller (10–15 µm in size) than the circulating endothelial cells but may express the same surface antigens [22].

The vascular endothelium is an unusual target of bacterial infections in humans [29]. The prototype bacteria that infect endothelial cells are the *Rickettsiae*; the species belonging to the typhus group (*Rickettsia typhi* and *Rickettsia prowazekii*) and spotted fever group (*Rickettsia rickettsii*, *R. conorii*, etc.) all have selective tropism for the endothelium [30]. Another member of the family *Rickettsiaceae*, *Orientia tsutsugamushi*, which causes scrub typhus, also has a predilection for endothelial cells [31]. Finally, *Bartonella* species invade microvascular endothelial cells before continuing on to invade erythrocytes [32]. All of these bacteria are strict or facultative (in the case of *Bartonella*) intracellular pathogens that are transmitted to humans via vectors, just like *Ca.* N. mikurensis. Amongst the A*naplasmataceae*, another recently recognized human pathogen, the *Ehrlichia muris*-like agent (EMLA), infected pulmonary vascular endothelial cells of experimentally inoculated laboratory mice [33] and white-footed mice experimentally infected with EMLA-infected *I. scapularis* nymphs [34]. In contrast, *A. phagocytophilum* and *E. chaffeensis*, although pathogenic for humans, are more distantly related to *Ca.* N. mikurensis and have leukocytes as their primary targets of infection [35]. However, human strains of *A. phagocytophilum* readily infect primate endothelial cells *in vitro* when transferred from infected tick or human cell cultures [21,36]. Similarly, the bovine pathogens *A. marginale* and *E. minasensis* infect mammalian endothelial cells *in vitro* when transferred from tick cells [17,21], although evidence for endothelial cell infection in cattle *in vivo* is inconclusive [37,38] or lacking [39]. *E. ruminantium*, the agent of the tick-borne disease heartwater that kills large numbers of domestic ruminants in Africa and some islands of the Caribbean, typically infects vascular endothelial cells as well as circulating neutrophils [25], and is closely related phylogenetically to *Ca.* N. mikurensis [26]. Another intraleukocytic veterinary pathogen, *E. canis*, has been reported to infect human microvascular endothelial cells *in vitro* [40] and pulmonary endothelial cells of an experimentally infected dog *in vivo* [41].

Our assertion that endothelial cells are targets of *Ca.* N. mikurensis infection provides an explanation for the puzzling observation that many patients have fallen ill during the winter months (in Sweden) when tick bites are extremely rare; all of these patients have had immune-suppressive medication, which suggests that the infection lay dormant and was re-activated when their immune defenses were impaired. This is reminiscent of Brill-Zinsser disease, the recrudescence of *R. prowazekii* infection in aged patients decades after the initial epidemic typhus infection [42]. The vascular endothelium provides an ideal niche for chronic persistent infections since endothelial cells have a very low turn-over with a daily replication rate of 0–1% [28]. Further support for the theory of chronicity of infection is that even immune-competent individuals harbour *Ca.* N. mikurensis DNA in their blood for many months [7,10].

According to the rules of bacterial nomenclature, bacterial species that have not yet been cultivated are given the “*Candidatus*” prefix. In view of our successful cultivation of six clinical isolates of *Ca.* N. mikurensis, we propose that this prefix be now removed, and that the bacterium be renamed *Neoehrlichia mikurensis*. To conclude, an important milestone has been reached – the cultivation of *Ca.* N. mikurensis in both tick cell lines and human endothelial cells. Furthermore, an essential gap in knowledge has been filled by establishing that the vascular endothelium is a target of neoehrlichial infection in humans. Both of these accomplishments, the cultivation of the bacterium and the identification of its cellular tropism should greatly facilitate future research on the pathogenic mechanisms used by this novel bacterium to cause infectious disease. Hopefully it will now be possible, using culture-derived bacteria, to develop diagnostic assays for monitoring antibody responses to the infection as well as for estimation of the epidemiology of neoehrlichial infections in different populations. Furthermore, the sequencing of the bacterial genome should be within reach now that the bacterium can be cultivated. An important limitation of studies on neoehrlichiosis is the lack of animal models since it is only human beings and dogs that become sick; rodents do not show overt signs of infection and are presumed to be healthy reservoirs [2]. For the time being, research on *N. mikurensis* will have to rely on patient samples and *in vitro* studies of infected tick and human cell lines.

## Materials and methods

### Infected tick material

Questing nymphal and adult *I. ricinus* ticks were collected in June and July of 2015 by blanket dragging at Koön and Klöverön outside Marstrand, on the west coast of Sweden. The ticks were washed in 70% ethanol, dried for a few seconds on filter paper and cut sagitally into two halves using a scalpel. The halves were separately homogenized in microcentrifuge tubes containing two 3.2 mm steel beads and distilled water (200 μl) in a Bullet Blender (Next Advance Inc, Troy, NY, USA) at speed 8 for 4 min. The homogenates were diluted in distilled water to a final volume of 400 μl and tested by *Ca.* N. mikurensis-specific real-time PCR as described below, either individually or in pools of four ticks. Alternatively, the extremities of the ticks were removed, the tick carcass was individually centrifuged at 8000*g* for 5 min, and clear haemolymph was collected and analysed by real-time PCR. PCR-positive haemolymph or tick homogenate was inoculated into cell lines.

Prevalence estimates were done on the ticks collected in June (*n* = 223; 94% nymphs, 6% adults) and July (*n* = 146; 95% nymphs, 5% adults). A first estimate of the *Ca*. N. mikurensis prevalence in the June collection was based on six pools of ticks containing four ticks each. Half of the pools were PCR-positive, yielding a minimal infection rate of 13%. Next, 33 ticks from the same collection were individually analysed by real-time PCR which resulted in a 9.1% infection rate (3/33). A higher infection rate of 15% was seen among the ticks collected in July: 11/71 individually analysed ticks were positive.

### Infected human material

Whole blood, plasma and buffy coats from EDTA-blood collected from patients who tested positive for *Ca.* N. mikurensis by real-time PCR (*n* = 7) were used for cultivation experiments (*n* = 6). All the patients were immunocompromised and presented with symptomatic febrile disease. Their blood specimens were also checked by 16S rRNA PCR [7] and resultant PCR products were sequenced to ensure there were no multiple infections or ambiguous DNA sequences. Clinical data are presented in Table 1; data pertaining to three of the patients have been published previously [11,43].

### Cell lines

Two embryo-derived tick cell lines were used: the *I. scapularis* cell line ISE6 at passage >82 [44] and the *I. ricinus* cell line IRE/CTVM20 at passage >210 [45], both obtained from the Tick Cell Biobank, UK. The following primary human cell lines were tested: primary dermal microvascular endothelial cells (ATCC PCS-110-010; ATCC, Manassas, VA, USA) and primary pulmonary artery endothelial cells (ATCC PCS-100-022). The tick cell line ISE6 was maintained in L-15B medium [46] supplemented with 10% heat-inactivated foetal bovine serum (FBS), 10% tryptose phosphate broth (TPB), 0.1% bovine lipoprotein (MP Biomedicals, Santa Ana, CA, USA), 2 mM l-glutamine, 100 U/ml penicillin and 100 μg/ml streptomycin (Invitrogen, Carlsbad, CA, USA). The tick cell line IRE/CTVM20 was maintained in a 1:1 mixture of L-15B medium supplemented as above, and unmodified L-15 (Leibovitz) medium supplemented with 20% FBS, 10% TPB, 2 mM l-glutamine and antibiotics as above. Both tick cell lines were grown in sealed, flat-sided culture tubes (Nunc, Roskilde, Denmark) in ordinary air at 28°C. The endothelial cells were seeded into 25 cm^2^ cell culture flasks with vented cap (TPP, Trasadingen, Switzerland) in vascular cell basal medium (ATCC PCS-100-030) supplemented with endothelial cell growth kit-BBE (ATCC PCS-100-040) containing 5 ng/ml vascular endothelial growth factor, 5 ng/ml epidermal growth factor, 5 ng/ml fibroblast growth factor, 15 ng/ml insulin-like growth factor, 10 mM l-glutamine, 0.75 U/ml heparin sulphate, 1 μg/ml hydrocortisone hemisuccinate, 2% FBS and 50 μg/ml ascorbic acid, and grown at 37°C in air supplemented with 5% CO_2_. No antibiotics were added to the medium.

### Inoculation of cell cultures with *Ca.* N. mikurensis

The various cell lines were inoculated with either of the following *Ca.* N. mikurensis-infected specimens: (1) tick haemolymph (pooled from 15 ticks, total volume 30–40 µl), (2) tick homogenates (10 ticks homogenized in 300 µl culture medium), (3) patient-derived whole blood (1 ml), and (4) patient-derived buffy coat admixed with plasma (1 ml). Cultures were allowed to proceed for between 2 weeks and 5 months. Cell culture supernatants as well as whole cell cultures were monitored for infectivity by real-time PCR as described below. All cell lines tested negative for *Mycoplasma spp.* by PCR [47].

### Passage of infection from tick cells onto fresh tick cells and cryopreservation

Infected tick cell cultures were harvested, divided into two and used to inoculate two new tubes containing fresh uninfected tick cells. Aliquots of infected tick cell cultures were cryopreserved with 10% dimethyl sulfoxide in a vapor-phase liquid nitrogen refrigerator as described previously [16].

### Transfer of infection from tick cells to endothelial cells

The protocol employed by Goodman et al. was used [48]. In short, infected tick cells were harvested (2 ml) and homogenized by passage through a 25-gauge needle three times, followed by centrifugation of the homogenate at 700*g* for 5 min to remove remaining intact cells and cellular debris. The supernatant was centrifuged at 1250*g* for 5 min and the resultant bacterial pellet was resuspended in 50 µl of endothelial cell medium and immediately inoculated into one of the primary endothelial cell lines.

### DNA extraction and *Ca.* N. mikurensis-specific real-time PCR

Extraction of DNA from tick and human specimens, as well as from whole cell cultures or cell culture supernatants, was performed using the Nucleic Acid Isolation Kit I (Roche Molecular Diagnostics, Pleasanton, CA, USA) in a MagNA Pure Compact Extraction Robot (Roche Molecular Diagnostics). DNA extracts were stored at −20°C unless the real-time PCR analyses were done immediately. The DNA extracts (undiluted, 1/10 and 1/100 dilutions) were analysed with a Taqman real-time PCR directed against a 169-bp segment of the *groEL* gene of *Ca.* N. mikurensis as previously described [7]. A synthetic plasmid containing the 169-bp sequence cloned into a pUC57 vector (Genscript, Piscataway, NJ, USA) was used both as a positive control and to estimate bacterial gene copy numbers. Samples that were positive by the *Ca.* N. mikurensis-specific *groEL*-based real-time PCR were re-analysed using a panbacterial PCR directed against the 16S rRNA gene and sequenced for confirmation [7].

### Imaging flow cytometry

*Ca.* N. mikurensis-infected and mock-infected tick cell cultures were harvested by pipetting, fixed and permeabilized using the BD cytofix and cytoperm kit (Becton Dickinson, San Jose, CA, USA) and then hybridized with the panbacterial 16S rRNA probe EUB338 [49] conjugated to Alexa Fluor 488 (Eurofins Genomics, Ebersberg, Germany) and with a newly designed 16S rRNA probe specific for *Ca.* N. mikurensis (CTATTTAAACTAGAGATCGAGAGAG) conjugated to Alexa Fluor 555 (Eurofins Genomics). Two complementary probes were used to control for non-specific hybridization: the non-EUB338 probe conjugated to Alexa Fluor 488 and the non-Neo probe conjugated to Alexa Fluor 555 (Eurofins Genomics). All probes were diluted in a hybridization buffer comprising 3.6 M NaCl, 80 mM Tris-HCl at pH 4.7 containing 30% formamide and 5% SDS. The cells were hybridized overnight with 10 ng/μl of each probe at 42°C, washed once using pre-heated hybridization buffer (42°C) minus formamide and SDS, followed by one PBS wash at room temperature. The DNA stain DRAQ5 (1 µM, Affymetrix eBioscience, San Diego, CA, USA) was added 10 min prior to sample acquisition without washing to label tick cell nuclei.

*Ca.* N. mikurensis-infected and mock-infected endothelial cells were harvested by flushing twice with 0.5 mM EDTA supplemented with 2% trypsin at 37°C. The endothelial cells were hybridized as described above for the tick cell lines using the EUB388 probe and the *Ca.* N. mikurensis-specific DNA probe. In addition, the cells were labelled using mAb anti-von Willebrand Factor conjugated to Alexa Fluor 405 (R&D Systems, Minneapolis, MN, USA), mAb anti-CD146 conjugated to PE or APC (Miltenyi Biotech, Bergisch Gladbach, Germany), and either of the nuclear stains DRAQ5 or DAPI (140 nM, Thermo Fisher Scientific, Waltham, MA, USA). Infected endothelial cells were also labelled using a 1/10 dilution of an immune serum from a neoehrlichiosis patient (SE13) [26] or a control serum from a healthy individual, at 37°C for 30 min. The cells were washed once, before the addition of affinity-purified FITC-labelled goat anti-human IgG antibody (Focus Diagnostics, Cypress, CA, USA) for 30 min at 37°C.

Images of 1000 cells were collected in the imaging flow cytometer (ImageStream®X Mark II; Amnis, Seattle, WA, USA) and analysed with IDEAS software 6.0. To determine what proportion of the cells were infected, a custom-made analysis strategy was employed. First, a mask defining the cytosol region was created. The area stained by the *Ca.* N. mikurensis-specific DNA probe was divided by the area of the cytosol mask, and the value was then multiplied by 100. The IDEAS feature used for calculation was as follows: Area_Threshold(M03, NEO, 30) / Area_Object(M04, BF, Tight) − Area_Threshold(M05, DRAQ5, 60) × 100. Results are expressed as the median feature value for 1000 cells from each culture.

### Giemsa-stained cytocentrifuge smears of infected cell lines

Infected tick cells were spun on to glass slides using a Shandon Cytospin cytocentrifuge (Thermo Fisher Scientific) at 1000 rpm for 5 min, fixed in 98% methanol for 10 min, and stained for 20 min using 10% Giemsa (Merck, Darmstadt, Germany) diluted in Sorensen’s phosphate buffer (0.2 M KH_2_PO_4_ and 0.2 M Na_2_HPO_4_ in distilled water, pH 6.7) and rinsed in distilled water. Endothelial cells were seeded on to Lab-Tek™ II Chamber slides (Nunc) placed in cell culture wells supplemented with 2 ml medium for one day, removed, fixed in 100% acetone for 10 min, and stained in Giemsa solution as described above.

### Circulating endothelial cells

Freshly isolated EDTA buffy coats from two patients with newly diagnosed neoehrlichiosis were incubated with Alexa Fluor 405-labelled mAb anti-von Willebrand Factor or APC-conjugated anti-CD146 mAb for 30 min at 4°C, fixed and permeabilized with BD Cytofix and Cytoperm kit (Becton Dickinson) and hybridized overnight using the Alexa Fluor 488-labelled *Ca.* N. mikurensis-specific DNA probe as described for the infected endothelial cell lines. Prior to analysis by imaging flow cytometry, the endothelial cell nuclei were stained using DRAQ5 or DAPI.
